# 1-aminocyclopropane-1-carboxylic acid (ACC) in plants: more than just the precursor of ethylene!

**DOI:** 10.3389/fpls.2014.00640

**Published:** 2014-11-11

**Authors:** Bram Van de Poel, Dominique Van Der Straeten

**Affiliations:** ^1^Department of Cell Biology and Molecular Genetics, University of Maryland, College ParkMD, USA; ^2^Laboratory of Functional Plant Biology, Department of Physiology, Ghent UniversityGhent, Belgium

**Keywords:** 1-aminocyclopropane-1-carboxylic acid (ACC), ethylene, conjugation, deaminase, transport, signaling

## Abstract

Ethylene is a simple two carbon atom molecule with profound effects on plants. There are quite a few review papers covering all aspects of ethylene biology in plants, including its biosynthesis, signaling and physiology. This is merely a logical consequence of the fascinating and pleiotropic nature of this gaseous plant hormone. Its biochemical precursor, 1-aminocyclopropane-1-carboxylic acid (ACC) is also a fairly simple molecule, but perhaps its role in plant biology is seriously underestimated. This triangularly shaped amino acid has many more features than just being the precursor of the lead-role player ethylene. For example, ACC can be conjugated to three different derivatives, but their biological role remains vague. ACC can also be metabolized by bacteria using ACC-deaminase, favoring plant growth and lowering stress susceptibility. ACC is also subjected to a sophisticated transport mechanism to ensure local and long-distance ethylene responses. Last but not least, there are now a few exciting studies where ACC has been reported to function as a signal itself, independently from ethylene. This review puts ACC in the spotlight, not to give it the lead-role, but to create a picture of the stunning co-production of the hormone and its precursor.

## THE DISCOVERY OF ACC

**FIGURE 1:**
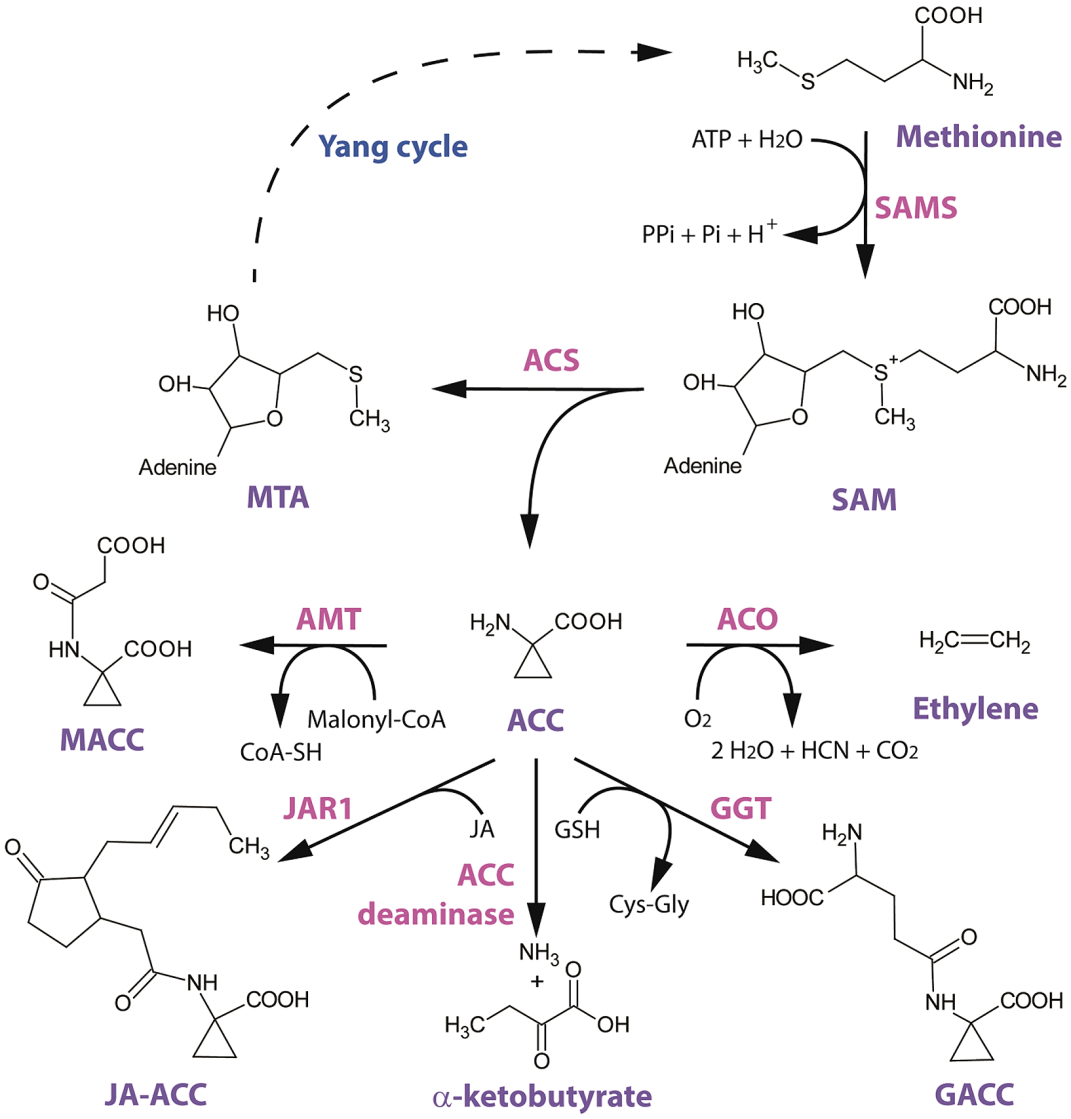
**Structural scheme of ethylene biosynthesis and 1-aminocyclopropane-1-carboxylic acid (ACC) conjugation/metabolism.** The amino acid methionine is converted to S-adenosyl-L-methionine (SAM) by SAM-synthetase (SAMS) with the requirement of ATP. The general precursor SAM is then converted to ACC by ACC-synthase (ACS). This reaction also involves the cleavage of 5′-methylthioadenosine (MTA), which is recycled back to methionine by the Yang cycle (dotted line indicates multiple enzymatic steps). ACC can be converted to ethylene by ACC-oxidase (ACO) in the presence of oxygen. ACC can also be converted to its major conjugate 1-malonyl-ACC (MACC) by the yet uncharacterized ACC-N-malonyl transferase (AMT) with the requirement of malonyl-Coenzyme-A. A second derivate of ACC is γ-glutamyl-ACC (GACC) which is formed by γ-glutamyl-transpeptidase (GGT) with the requirement of glutathione (GSH). Another novel derivate of ACC is jasmonyl-ACC (JA-ACC), which is formed by jasmonic acid resistance 1 (JAR1). ACC can also be metabolized by the bacterial (and plant) ACC deaminase into ammonium and α-ketobutyrate.

The discovery of ethylene as a plant growth regulator can be attributed to the work of the Russian scientist Neljubov (1901). He reported that dark-grown pea seedlings showed a reduced hypocotyl growth in combination with an exaggerated hypocotyl bending when exposed to illumination gas (Neljubov, 1901). Neljubov (1901) could pinpoint ethylene gas as the active component that caused dark-grown pea seedlings to bend, by flowing the illumination gas over several filters prior to exposing the seedlings. This typical ethylene response of dark-grown seedlings was later defined as the triple response: (1) shortening of the hypocotyl and roots, (2) radial swelling of the hypocotyl, and (3) the exaggeration of the apical hook (Knight et al., 1910). In 1934, conclusive evidence that ethylene is a natural product from plants, was presented by the English scientist (Gane, 1934). It took another 30 years before the primary steps of the ethylene biosynthesis pathway were elucidated (see **Figure 1**). Lieberman and Mapson (1964) first reported that ethylene could be produced from the amino acid methionine, taking advantage of the high rates of ethylene production from apples for their experimental work (**Figure 1**). 13 years later, Adams and Yang (1977) made tremendous progress in understanding the biosynthesis pathway of ethylene, when they discovered that S-adenosyl-L-methionine (SAM) was an intermediate between methionine and ethylene. Yang and co-workers also showed that 5′-methylthioadenosine (MTA) was formed as a by-product from SAM and that MTA could be recycled back to methionine (Murr and Yang, 1975). The elaboration of the different reaction steps of the methionine cycle in plants, now often referred to as the Yang-cycle, was mainly inspired by the biochemical similarities between the plant pathway and the methionine salvage cycle which was already known for prokaryotes, yeast, and mammalians. An-up-to-date overview of the methionine and SAM metabolism in plants is given by Sauter et al. (2013). The major discovery that made the methionine cycle in plants unique from all other organisms, was the characterization of 1-aminocyclopropane-1-carboxylic acid (ACC) as the intermediate between SAM and ethylene (Adams and Yang, 1979). Adams and Yang (1979) were able to identify ACC as the precursor for ethylene by feeding experiments on apple tissue, using radio-labeled methionine. Upon incubation of apple disks, they observed a shift from ethylene production in air, toward an unknown compound that was retained in the tissue when treated with nitrogen (lack of oxygen inhibits oxidation of ACC toward ethylene). By using a pH-dependent ion mobility assay, they could characterize this unknown component as an amino acid. Subsequently, the component was identified as ACC, using co-migration of synthetic ACC for both paper-chromatography and paper-electrophoresis (Adams and Yang, 1979). They further showed that the conversion of radioactively labeled methionine toward ethylene decreased when unlabeled ACC was supplemented, yet the conversion of labeled ACC to ethylene was almost not affected when unlabeled methionine was supplemented, suggesting that externally supplied ACC is in fact used to produce ethylene. Additional evidence for ACC being the intermediate precursor between SAM and ethylene was obtained by treating apple tissue with [S]-trans-2-amino-4(2′-aminoethoxy)trans-3-butenoic acid, also known as AVG (2-amino-ethoxy-vinylglycine), a pyridoxal-5′-phosphate (PLP or vitamin B_6_) dependent enzyme inhibitor, which was later known to inhibit the enzymatic conversion of SAM toward ACC. The identification of ACC as the precursor of ethylene was a major breakthrough in the understanding of the ethylene biosynthesis pathway in plants, and was part of the foundation for many new discoveries in the field of ethylene biology.

## ACC AND ETHYLENE BIOSYNTHESIS

As mentioned above, ACC is produced from SAM, releasing MTA. This reaction is catalyzed by the enzyme ACC-synthase (ACS; Boller et al., 1979). ACS is a member of the PLP-dependent enzymes, which use vitamin B6 as a co-factor for its enzymatic function. ACS was localized in the cytosol by activity assays on extracts retrieved after differential centrifugation (Boller et al., 1979). ACS genes were first characterized in zucchini by Sato and Theologis (1989) and in tomato by Van Der Straeten et al. (1990). ACS is encoded by a multigene family of 12 members in *Arabidopsis*, eight of which encode functional ACC synthases [ACS2 (named ACS1 in Van Der Straeten et al., 1992), ACS4-9, ACS11]. In addition, there is one inactive isoform (*AtACS1*) and one pseudogene (*AtACS3*; Yamagami et al., 2003). ACS was found to form functional dimers of which the 3D structure was determined by Capitani et al. (1999). The formation of heterodimers increases the structural and functional complexity of the ACS protein family (Tsuchisaka et al., 2009). The large *ACS* gene family displays a tissue-specific and differential expression pattern in *Arabidopsis* (Tsuchisaka and Theologis, 2004). Using single and multiple *acs* knock-out mutants, it was demonstrated that there are specific developmental and physiological roles for individual members of the *ACS* gene-family, but also that there is a complex combinatorial interplay amongst them (Tsuchisaka et al., 2009). A diverse group of internal and external signals modulate the level of ethylene biosynthesis in numerous plant species, acting at the level of *ACS* gene expression. These inducers include auxin, cytokinin, brassinosteroids, ethylene, copper, mechano-stimuli, ozone, pathogens and wounding (Van Der Straeten et al., 1992; Rodrigues-Pousada et al., 1993; Botella et al., 1995; Cary et al., 1995; Liang et al., 1996; Vahala et al., 1998; Woeste et al., 1999).

Three types of ACS proteins are recognized, based on their *C*-terminal structure. Type I ACS proteins contain in their *C*-terminal domain one putative calcium-dependent protein kinase (CDPK) phosphorylation target site and three mitogen-activated protein kinase (MAPK) phosphorylation sites (Yoon and Kieber, 2013). Type II ACS proteins only contain the MAPK phosphorylation sites, while type III ACS do not contain any phosphorylation sites (Yoon and Kieber, 2013). These post-translational phosphorylation sites play an important role in the stability of the ACS protein (Chae and Kieber, 2005). Both in *Arabidopsis* (Chae et al., 2003; Kim et al., 2003; Liu and Zhang, 2004; Wang et al., 2004; Yoshida et al., 2005, 2006; Joo et al., 2008; Christians et al., 2009; Lyzenga et al., 2012) and in tomato (Tatsuki and Mori, 2001; Kamiyoshihara et al., 2010) it was shown that differential phosphorylation of certain ACS members directed the protein for proteasomal degradation. Protein stability of certain ACS members is further regulated by the protein phosphatase 2A (PP2A; Skottke et al., 2011) and PP2C (Ludwikow et al., 2014), demonstrating a complex balance between phosphorylation and dephosphorylation to secure protein activity and stability.

The second ethylene biosynthesis protein is ACC-oxidase (ACO), which converts ACC to ethylene in the presence of oxygen. It took a long time before ACO activity could be demonstrated *in vitro*. The key aspect in isolating ACO was the addition of ascorbic acid (vitamin C) to the extraction media, as was first reported by Ververidis and John (1991) who isolated ACO from melon tissue and quantified *in vitro* ACO activity. Although the exact role of ascorbic acid for protein stability/activity remained uncertain for a long time (Rocklin et al., 2004), it was recently clarified that ascorbic acid participates in the ring opening of ACC, by providing a single-electron to the active site (Murphy et al., 2014). This catalytic reaction releases ethylene and a cyanoformate ion [NCCO_2_]^-^, which is subsequently decomposed into CO_2_ and CN^-^ (Murphy et al., 2014). The reactive cyanide (CN^-^) is subsequently detoxified by β-cyanoalanine synthase to produce β-cyanoalanine (Miller and Conn, 1980). ACO belongs to the superfamily of dioxygenases that require iron (Fe^2+^) as co-factor and bicarbonate as activator (Dong et al., 1992; Zhang et al., 2004). The subcellular localization of ACO remains vague, as some studies localize ACO in the cytosol (Reinhardt et al., 1994; Chung et al., 2002; Hudgins et al., 2006), while other localize ACO at the plasma membrane (Rombaldi et al., 1994; Ramassamy et al., 1998). Although the ACO protein sequence does not contain any predicted transmembrane domains, it is still possible that the protein associates with the plasma membrane via (in)direct interactions. ACO is also encoded by a multigene family of five members in *Arabidopsis* [ACO1, 2, 4, At1g12010 (AOC3) and At1g77330 (ACO5)]. Expression of different members of the tomato ACO family in *Escherichia coli* showed that each isoform had a specific *in vitro* enzyme activity (Bidonde et al., 1998).

It is well accepted that ACS is the rate limiting step of ethylene biosynthesis in plants (Yang and Hoffman, 1984) although there are examples where ACO is the rate limiting step, e.g., during post-climacteric ripening of tomato fruit (Van de Poel et al., 2012). Three ACO genes are also auto-regulated by ethylene in *Arabidopsis* (De Paepe et al., 2004). This might suggest that the regulation of ACO expression and/or activity is more complex than anticipated. There are some hints for a putative post-transcriptional and/or post-translational regulatory mechanisms of ACO as suggested by Dilley et al. (2013) and as investigated through mathematical modeling by Van de Poel et al. (2014a).

Both ACS and ACO are two well-studied enzymes that exclusively participate in the ethylene biosynthesis pathway. Both proteins are well characterized, but many more questions remain unanswered. While the post-translational regulation of ACS has been revealed, the biochemical and mechanistic details of this protein modification are still unclear. Transcriptional and functional characterization of the different *ACS* gene-family members has shone light on the combinatorial interplay, nevertheless, much more work is needed to elucidate the exact role of each isoform and how they interact with each other. Much less is known about the post-translational regulation and combinatorial interplay of ACO. A lack of genetic studies focusing on ACO, raises the question whether or not ACO shares a similar structural, biochemical and post-translational complexity as ACS.

## ACC AS A PIVOTAL MOLECULE: ACC CONJUGATES AND THE CONTROL OF ETHYLENE BIOSYNTHESIS

1-Aminocyclopropane-1-carboxylic acid is best known as the direct precursor of ethylene in the ethylene biosynthesis pathway. However, there also exist three different conjugates of ACC, suggesting that the biochemical regulation of the available ACC pool is more complex than anticipated, which in turn can possibly affect the eventual levels of the plant hormone ethylene.

Shortly after the identification of ACC as the intermediate between SAM and ethylene in the ethylene biosynthesis pathway, a first conjugated form of ACC, called malonyl-ACC (MACC), was discovered by Amrhein et al. (1981) in buckwheat seedlings and by Hoffman et al. (1982) in wheat leaves. MACC is formed by ACC-N-malonyl transferase (AMT) which was purified from tomato extracts (Martin and Saftner, 1995), although not structurally characterized. It was shown that the conjugation of ACC into MACC was stimulated by ethylene in preclimacteric tomatoes (Liu et al., 1985a), grapefruit flavedo (outer peel; Liu et al., 1985b) and tobacco leaves (Philosoph-Hadas et al., 1985), indicative for a feedback control of ethylene biosynthesis. Martin and Saftner (1995) also showed that the activity of AMT was ethylene inducible and that its activity correlated with the increase in ethylene production during climacteric ripening of tomato (Martin and Saftner, 1995). The exact amino acid and gene sequences of AMT are not yet known, and no putative *AMT* gene is annotated in the *Arabidopsis* genome, limiting more in-depth genetic and molecular studies. Because MACC does not participate in any other known biological conversions, MACC formation might be a mechanism to control the available ACC pool. This hypothesis was further strengthened by the observation that MACC could be translocated from the cytosol into the vacuole (and back) by ATP-dependent tonoplast carriers (Bouzayen et al., 1988, 1989; Tophof et al., 1989). Nonetheless, the reconversion of MACC toward ACC by an unknown MACC-hydrolase was reported twice in literature (Jiao et al., 1986; Hanley et al., 1989). The ability to hydrolyze MACC back into ACC and the ability to ‘store’ MACC in the vacuole is an interesting mechanism to regulate the cellular availability of MACC. Moreover, because MACC has no other biochemical role besides being an ACC conjugate, the regulation of MACC levels can also affect the available pool of ACC and possibly ethylene production levels. This hypothesis was investigated by *in silico* mathematical modeling, showing that the reconversion of MACC to ACC could have a potential stimulating effect on ethylene production during climacteric fruit ripening of tomato (Van de Poel et al., 2014a).

A second important derivative of ACC is γ-glutamyl-ACC (GACC), which was discovered in crude tomato extracts of ACC-N-malonyltransferase (Martin et al., 1995). These crude protein extracts were able to form a new ACC derivative, which could be identified as GACC (Martin et al., 1995). GACC is formed by the reaction of ACC with the tripeptide glutathione (GSH) by a γ-glutamyl-transpeptidase (also called γ-glutamyl-transferase, GGT; Martin et al., 1995; Martin and Slovin, 2000). While a first report, based on *in vitro* studies, stated that GACC was the most abundant ACC derivative in tomato (Martin and Saftner, 1995; Martin et al., 1995), another study found that MACC was the most abundant ACC derivative *in vivo* in tomato fruit during climacteric ripening (Peiser and Yang, 1998). The *Arabidopsis* genome contains four genes (*GGT1*-*4*), of which only GGT1 and GGT2 are catalytically active (Martin et al., 2007). Both *GGT1* and *GGT2* are co-expressed predominantly in rapidly growing tissue, and are localized extracellularly, which raised the question about the role of extracellular GACC (Martin et al., 2007). A knock-out mutant of *GGT1* shows rapid senescence, *while GGT3* knock-outs have a reduced rosette size and silique number (Martin et al., 2007). The effect of GACC formation by GGT on ACC availability and possibly ethylene biosynthesis remains to be investigated.

A third derivative of ACC is jasmonyl-ACC (JA-ACC). This molecule was discovered by screening for amino acid conjugates of JA, using GC-MS (Staswick and Tiryaki, 2004). Four amino acid conjugates of JA (JA-Ile, JA-Val, JA-Leu, and JA-Phe) were quantified in *Arabidopsis* tissue (Staswick and Tiryaki, 2004). Interestingly, the same authors also demonstrated that JA forms a conjugate with ACC (JA-ACC) in *Arabidopsis* leaves (Staswick and Tiryaki, 2004). Recombinant JAR1 enzyme was found to be able to form JA-ACC *in vitro*. Strangely, levels of JA-ACC were higher in the leaves of *jar1* mutants compared to wild-type plants. It was also shown that JA-ACC inhibits root growth in *Arabidopsis*. Elegant genetic experiments with JA signaling mutants (*coi1-35*) showed that the JA-ACC-induced root inhibition was independent of JA signaling. Furthermore, the ethylene signaling mutant *etr1-1* and the double mutant *etr1-1 jar1-1* were insensitive to the JA-ACC treatment and displayed no inhibition of root growth, indicating that JA-ACC acts via the ethylene signaling pathway (Staswick and Tiryaki, 2004). Most likely the ACC moiety of JA-ACC is responsible for an increase in ethylene production which results in the root growth inhibition response. These experiments suggest that JA-ACC might serve as a pivotal molecule which can function as a modulator of the hormonal cross-talk between the ethylene and jasmonic acid pathway, although the exact molecular and biochemical mechanism of JA-ACC function remains unclear.

The three above-mentioned derivatives of ACC (MACC, GACC, and JA-ACC) are perhaps not the only ones. Future metabolic studies might reveal additional conjugates of ACC. Nonetheless, these three derivatives can potentially play an important role in the regulation of the pool of available ACC, which in turn can affect eventual ethylene production levels, with physiological and developmental consequences. Genetic perturbations of the formation of ACC derivatives could be a useful tool to unravel their exact roles. In addition, a more detailed structural and biochemical characterization of the enzymes involved in the formation of ACC derivatives is essential.

## ACC DEAMINASE AND PLANTS

As mentioned above, plants possess several mechanisms to control their pool of ACC, for example by converting it to ethylene or to conjugates like MACC, GACC, or JA-ACC. Another unique way to metabolize ACC, is the deamination of ACC. ACC deaminase was first discovered in bacteria. Some plant growth-promoting bacteria are capable of processing the plant-borne ACC by converting it into ammonia and α-ketobutyrate using the enzyme ACC deaminase (Honma and Shimomura, 1978). ACC deaminase was retrieved in for example, *Pseudomonas* sp. strain ACP (Honma and Shimomura, 1978), *Pseudomonas chloroaphis* 6G5 (Klee et al., 1991), *Pseudomonas putida* GR12-2 (Jacobson et al., 1994) and *Pseudomonas putida* UW4 (Hontzeas et al., 2004).

The bacterial ACC deaminase is a PLP-dependent enzyme with a rather low affinity for ACC (the reported Km value is 1.5–15 mM; Glick et al., 2007). Nonetheless, relatively low concentrations of ACC (100 nM) can already induce the expression of the ACC deaminase gene (*acdS*), but so do other amino acids like L-alanine, DL-alanine, and DL-valine (Jacobson et al., 1994). *acdS* expression is also under the regulation of the nitrogen fixation (*nif*) promotor of some Rhizobia, linking ACC deaminase with nodule formation (Nukui et al., 2006; Nascimento et al., 2012).

Plant growth-promoting bacteria that harbor ACC deaminase must interact with the root environment in order to access plant-produced ACC. It was shown that root exudates contain certain amounts of ACC, which might attract ACC deaminase containing bacteria and establish the rhizosphere interaction (Penrose et al., 2001). It has been proposed that bacterial ACC deaminase can reduce the endogenous ethylene levels of plant roots by limiting the amount of available ACC, which will in turn prevent ethylene-induced root growth inhibition, and thus promote plant growth (Glick et al., 1998, 2007; Glick, 2014). Another model proposes that plant growth-promoting bacteria produce IAA which can be taken up by the plant, and can induce the expression of *ACS*, resulting in an increase in ACC production, providing a nitrogen supply for the bacteria (Glick et al., 1998, 2007; Glick, 2014).

There are many beneficial effects of ACC deaminase containing bacteria on plant growth, particularly in relation to stress tolerance. For instance, ACC deaminase containing bacteria can reduce stress susceptibility of plants during flooding (Barnawal et al., 2012; Li et al., 2013), drought (Mayak et al., 2004a), salinity (Mayak et al., 2004b; Nadeem et al., 2007, 2010), flower senescence (Nayani et al., 1998; Ali et al., 2012), metal pollution (Glick, 2010), organic pollution (Gurska et al., 2009) and pathogens (Glick, 2014 and references therein). In addition, it has been reported that the presence of ACC deaminase can increase the symbiotic performance of Rhizobial strains (Ma et al., 2003).

Hence, bacterial ACC deaminase is also used as a biotechnological tool to control endogenous ACC levels and consequently lower ethylene production in plants. Transgenic plants overexpressing bacterial ACC deaminase were shown to be more resistant to growth inhibition when confronted with fungal pathogens (Lund et al., 1998; Robison et al., 2001), salt stress (Sergeeva et al., 2006), and metals (Grichko et al., 2000; Nie et al., 2002).

Plants themselves also contain a homolog of the bacterial ACC deaminase. In *Arabidopsis*, it was demonstrated that the previously known enzyme D-cysteine desulfydrase also possesses ACC deaminase activity (McDonnell et al., 2009). Antisense lines showed a decreased ACC deaminase activity, an increased sensitivity to ACC and produced more ethylene (McDonnell et al., 2009). These results indicate that the plant-specific ACC deaminase might be another metabolic shunt regulating ACC levels and ethylene production in plants.

## ACC TRANSPORT AND ITS ROLE DURING ROOT STRESS

Besides the biosynthesis, conjugation, and catabolism of a hormone, short or long range transport is another important aspect to regulate proper dosage of a hormonal signal within an organism. Often, hormones are synthesized at one site and transported to another site for their action. Hormonal transport from one cell to another is an advanced process, that facilitates tissue specific or long-distance physiological processes or stress responses. Because ethylene is a gaseous molecule, it can freely diffuse from one cell to a neighboring cell, evoking mainly local responses. The presence of aerenchyma or large intercellular voids facilitates rapid long-distance transport of ethylene gas in plant organs. But long-distance ethylene responses can also be achieved by transport of its precursor ACC. Often, but not always, ACC is transported from the roots to the shoot, when the roots are exposed to stress (McManus, 2012). Yet, local ACC transport between cells of the same tissue type and intracellular transport is also possible, illustrating the molecular complexity of ACC transport.

**FIGURE 2:**
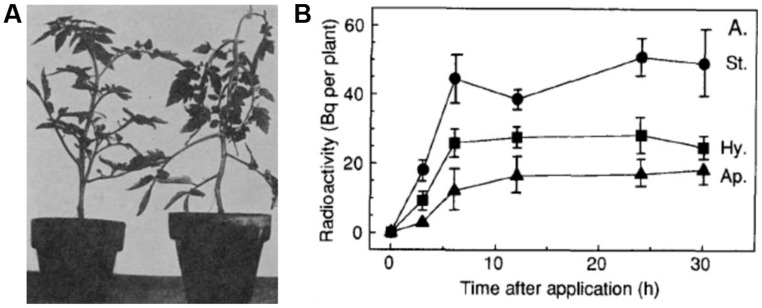
**1-Aminocyclopropane-1-carboxylic acid transport in plants. (A)** The epinastic response of tomato plants treated with ethylene gas as first observed by Doubt (1917). Figure reproduced from Doubt (1917). **(B)** ACC translocation via the phloem was demonstrated by measuring total radioactivity over time in the stem (St.), hypocotyl (Hy.), and apex (Ap.) after the foliar application of radioactive ACC to the oldest leaf (leaf 1) of 21 day old cotton plants. Figure reproduced from Morris and Larcombe (1995).

One of the best characterized ACC transport systems is the translocation of ACC from the roots to the shoots of tomato plants suffering from flooding or root hypoxia. A lack of oxygen in the rhizosphere will induce the expression of *ACS* in the roots (Olson et al., 1995; Shiu et al., 1998) resulting in an increased ACS activity (Bradford et al., 1982; Wang and Arteca, 1992). The excess of ACC in the roots is not converted to ethylene due to a lack of oxygen and the absence of ACO in the roots. Rather, ACC is loaded into the xylem and transported to the shoots (Bradford and Yang, 1980). Once arrived at the shoots, ACC is converted into ethylene by ACO, which is already present in the leaves (English et al., 1995). In tomato, root hypoxia will result in an epinastic response of leaves due to an increased ethylene production (**Figure 2A**; Doubt, 1917; Jackson and Campbell, 1976). Differential expression of both *ACS* and *ACO* during hypoxia was also observed in *Arabidopsis* (Peng et al., 2005), sunflower seedlings (Finlayson et al., 1991), maize (Atwell et al., 2006; Geisler-Lee et al., 2010), and rice (Zarembinski and Theologis, 1993, 1997; Van Der Straeten et al., 1997, 2001; Zhou et al., 2001). Interestingly, long-distance transport of ACC has also been suggested to occur during other root stress conditions as for example drought (Davies et al., 2000; Sobeih et al., 2004; Skirycz et al., 2011), rehydration after drought (Tudela et al., 1992), nutrient stress (Lynch and Brown, 1997) and salinity (Feng and Barker, 1992; Ghanem et al., 2008).

Another long-distance ACC transport system is achieved via the phloem. Foliar applied radioactive ACC was found to be transported via the phloem to other aerial parts in tomato (Amrhein et al., 1982) and in cotton plants (Morris and Larcombe, 1995; **Figure 2B**). It should be noted that the foliar applied ACC was also rapidly converted into MACC, which was not found to be transported via the phloem (Morris and Larcombe, 1995). This immobility of MACC is in accordance with the earlier observations that MACC is actively transported from the cytosol into the vacuoles, where it could be subsequently stored (Bouzayen et al., 1988, 1989; Tophof et al., 1989). Interestingly, there could be a link between phloem transport of ACC and the Yang cycle. Pommerrenig et al. (2011) showed that Yang cycle genes were specifically expressed in phloem, indicating that recycling of MTA toward SAM is preferentially carried out in this tissue. Perhaps MTA recycling is stimulated by high rates of ACC synthesis in phloem cells, or the recycled SAM forms a pool for phloem-specific ACC production. In roots, spatiotemporal gene expression profiling demonstrated that different *ACS* isoforms are expressed (but not exclusively) in the vascular tissue (Brady et al., 2007; Dugardeyn et al., 2008). Moreover, the loading of ACC to this tissue could affect the homeostasis of SAM and consequently, polyamines (Pommerrenig et al., 2011).

The exact molecular mechanism by which ACC is loaded into the xylem and/or the phloem and subsequently transported throughout the plant is still unknown, but is an important element in our understanding of long-distance ethylene signaling via ACC and how plants deal with different root and leaf stress conditions.

Long- or medium–long-distance ACC transport was also observed (or speculated) during different developmental processes. Tissue specific gene expression profiling of maize root cells showed that there are differences between *ACS* and *ACO* expression patterns (Gallie et al., 2009). *ACO* was predominantly expressed in the protophloem sieve elements and the companion cells, while *ACS* was expressed only in the root cortex. This discrepancy led the authors to hypothesize that ACC could be transported from the site of synthesis to the site of consumption, in order to ensure the ethylene production levels observed (Gallie et al., 2009). Differences in *ACS* and *ACO* expression patterns predicted *in silico* in *Arabidopsis* roots support the same hypothesis (Dugardeyn et al., 2008). A similar reasoning was made by Jones and Woodson (1997, 1999), who observed differences in *ACO* and *ACS* transcripts in different cell-types of carnation flower. They also postulated that ACC transport from sites with a high *ACS* expression (in e.g., petals and styles) secured the ability of ACO to produce ethylene in cells with a high *ACO* expression (for example the ovaries; Jones and Woodson, 1997, 1999).

Of course one should take into account that gene expression levels not always reflect actual protein levels, and that post-translational modifications can play an important role in protein stability and activity. A targeted metabolomics and proteomics study by Van de Poel et al. (2014b) investigated the tissue specificity of the ethylene biosynthesis pathway in tomato fruit. They observed that the pericarp tissue produced the highest amount of ethylene (and high ACO activity), while the pericarp had the lowest ACS activity and ACC content compared to other tissues. Perhaps ACC is transported from neighboring tissues with a high ACS activity or ACC content such as the locular gel, toward the pericarp in order to secure high rates of ethylene production during climacteric ripening of tomato (Van de Poel et al., 2014b).

Besides long-distance, short-distance intracellular transport of ACC was also observed in barley and wheat mesophyll cells (Tophof et al., 1989) and maize mesophyll cells (Saftner and Martin, 1993). ACC is transported across the tonoplast by carriers that rely on an electrochemical potential gradient of protons, and which are stimulated by the supplementation of ATP (Saftner and Martin, 1993). This intracellular compartmentalization of ACC allows the plant to precisely regulate the cellular pool of ACC, possibly also affecting ethylene biosynthesis.

Clearly, more research is needed to further unravel the exact molecular and biochemical mechanisms which assure intracellular, inter- and intra-tissue and long-distance ACC transport in plants, and their corresponding physiological effects.

## ACC AS A SIGNALING MOLECULE

1-Aminocyclopropane-1-carboxylic acid holds a key position in many physiological processes as it is the direct precursor in the biosynthesis of ethylene. A balanced supply and consumption of ACC is essential to achieve the necessary production level of ethylene within a given spatial and temporal context. As shown above, the pool of ACC is regulated by a complex interaction of production, consumption, modification, and transport. Interestingly, recent findings have suggested a perhaps even more important role for ACC, as a signaling molecule independent from ethylene (Yoon and Kieber, 2013).

**FIGURE 3:**
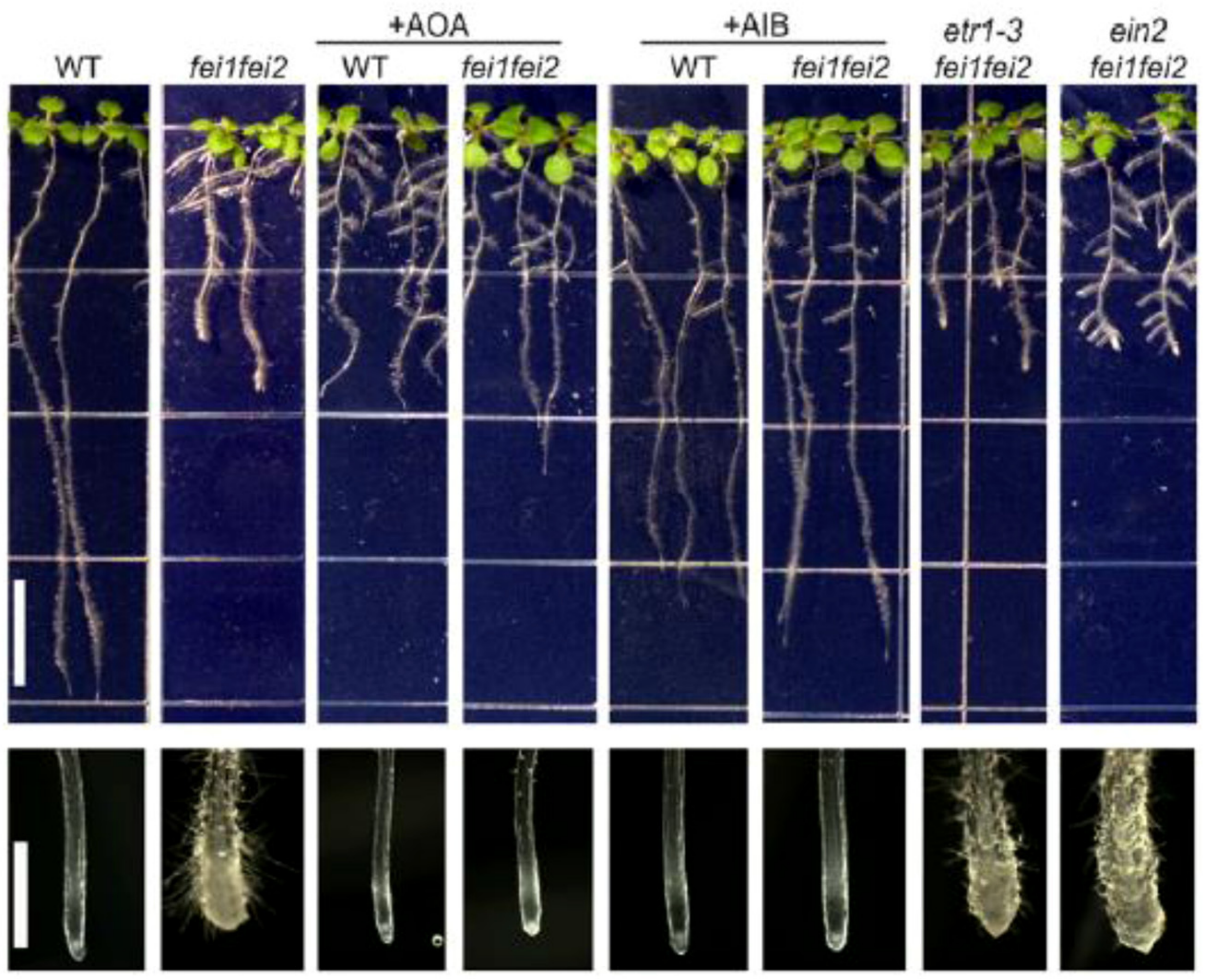
**Role of ACC/ethylene on the *fei* phenotype.** Root phenotypes of seedlings grown on MS medium containing 0% sucrose for 4 days and then transferred to MS medium containing 4.5% sucrose, or additionally supplemented with AOA (0.375 mM) or AIB (1 mM) as indicated. Note that the distribution of lateral roots in the *fei1 fei2* mutants in the presence of high sucrose is variable; the architecture of the *fei1 fei2 ein2* triple mutant is not substantially different from that of the *fei1 fei2* parent. The close-ups of the root tips clearly show the typical swelling of the *fei1 fei2* mutant. (Bar = 1 cm). Figure reproduced from Xu et al. (2008).

A first report by Xu et al. (2008) investigated the role of ACC signaling in relation with FEI1 and FEI2, which are leucine-rich repeat receptor-like kinases. The *fei1 fei2* mutant displays a severe defect in anisotropic root growth due to a reduced cellulose microfiber content in the cell wall at the root tip (**Figure 3**). The *fei1 fei2* phenotype can be reversed by the application of ethylene biosynthesis inhibitors, but not by ethylene signaling inhibitors. The application of both aminooxy-acetic acid (AOA) or α-aminoisobutyric acid (AIB) specifically inhibits ethylene biosynthesis and can reverse the phenotype of the *fei1 fei2* mutant (**Figure 3**). AOA is an inhibitor of PLP-dependent enzymes, and will affect the activity of ACS resulting in a reduced ethylene production. AIB on the other hand is a structural analog of ACC and acts as a competitive inhibitor of ACC preventing ethylene production at the level of ACO. Ethylene signaling inhibitors such as 1-methylcyclopropane (1-MCP) and silver ions, did not affect the *fei1 fei2* phenotype (**Figure 3**). Similarly, genetics showed that the *fei1 fei2* mutant crossed with *etr1-3* (a mutation in the ethylene receptor causing severe ethylene insensitivity), nor *ein2-50* (a central regulator of ethylene signaling causing ethylene insensitivity) could reverse the phenotype. All together this study showed that the typical *fei1 fei2* phenotype was not affected by ethylene signaling, but could be reversed by ethylene biosynthesis inhibitors. This suggests that the signal reversing the *fei1 fei2* phenotype originated independent from ethylene signaling, involved ACS and is possibly ACC itself (Xu et al., 2008).

A second report by Tsang et al. (2011) linked ACC signaling with cell elongation and cell wall composition of roots. Specific ethylene biosynthesis inhibitors [AVG, AOA, and 2-anilino-7-(4-methoxyphenyl)-7,8-dihydro-5(6H)-quinazolinone (7303)] could reverse the inhibition of root cell expansion which was induced by an isoxaben treatment (a cellulose biosynthesis inhibitor causing cell wall stress). Similarly, as observed by Xu et al. (2008), an ethylene signaling inhibitor (silver ions) could not reverse the isoxaben-induced reduction in root cell elongation. Also, the *ein3 eil1* ethylene insensitive mutant responded to ACC and isoxaben, providing genetic evidence of an ACC signaling mechanism independent of ethylene signaling. They also showed that the application of ACC without isoxaben, inhibited root cell elongation and was partially ethylene-dependent and partially ethylene-independent. Altogether, their results demonstrate that monitoring of cell wall integrity requires an ACC sensing/signaling mechanism, which can result in a reduction of root cell elongation, when disrupted. In addition, Tsang et al. (2011) showed that this inhibition of root cell elongation required auxin and reactive oxygen species (ROS) signaling, downstream of ACC signaling.

In a third report (Tsuchisaka et al., 2009) an octuple *acs* mutant was made to study the interplay between different ACS isoforms. The octuple line was created by introduction of two amiRNA lines (*ACS8* and *ACS11*) into the hexuple mutant *acs2,4,5,6,7,9* creating an octuple mutant line with complete or severe inhibition of ACS function. The lines that showed a very strong silencing of *ACS8* and *ACS11*, displayed embryo lethality. This suggests that ethylene biosynthesis (or ACC biosynthesis) is essential for *Arabidopsis* viability, while this is not the case for the single (*ctr1* and *ein2*) and double (*ctr1 ein2*) ethylene signaling mutants (Kieber et al., 1993; Roman et al., 1995; Alonso et al., 1999). This phenotypic discrepancy between ethylene biosynthesis and signaling once more suggests that ACC can acts as a signaling molecule itself, independent from ethylene, at least during embryo development and *Arabidopsis* viability. In addition, Tsuchisaka et al. (2009) characterized a wide variety of physiological and developmental processes of their single and multiple *acs* knock-out lines, and observed many phenotypes which were similar as for previously described ethylene signaling mutants. But interestingly they also observed several phenotypes like reduced branching, which were not observed in ethylene-insensitive mutants. These discrepancies could again be caused by ACC acting as a signaling molecule. However, it is also possible that individual ACS members have unique roles in developmental processes, and that knocking-out multiple ACS members might result in pleiotropic effects irrelevant to ACC or ethylene metabolism. Finally, it must be noted that such a severe genetic interference in ACC metabolism might also affect upstream SAM or MTA levels or the pool of downstream ACC conjugates, which in turn could signal themselves and affect cell physiology to contribute to the phenotypic differences observed.

All together these reports suggest a role for ACC as a signaling molecule to regulate plant development and growth, independent from ethylene. The exact molecular mechanism by which ACC signaling operates, and whether or not there is an ACC receptor and downstream signaling components, remains to be investigated. Future biochemical studies with specific ethylene biosynthesis and signaling inhibitors, in combination with genetics to create higher order ethylene biosynthesis/signaling mutants (like *etr1ers1etr2ein4ers2ctr1ein2* or multiple *aco* knock-outs), could shed light on the role of ACC as a signaling molecule. It also still needs to be elucidated whether this unique title of “signaling molecule” is to be awarded to ACC, or rather to one if its (unknown) downstream derivatives.

## CONCLUSION

Over the years, a lot of work has been done on ACC since its discovery in 1979, and it has become clear that ACC is more than just the precursor of ethylene. Its role in ethylene biosynthesis is well characterized, although there are still many questions concerning the two unique proteins that are associated with ACC in ethylene biosynthesis: ACS and ACO. Pioneering work on the characterization of post-translational modifications and the combinatorial interplay of ACS isoforms, has opened our eyes to the complex regulation of ethylene biosynthesis at the protein level. More mechanistic details are to be uncovered, probably including a complex post-translational control of ACO. Furthermore, ACC is conjugated into MACC, GACC, and JA-ACC. These derivatives are an elegant biochemical shunt to regulate the pool of ACC available for ethylene production, although it remains rather speculative what the exact biological roles are for these ACC conjugates. A better characterization of the participating enzymes is necessary to further elucidate the importunateness of ACC derivatives. Furthermore, ACC can also be used by bacterial (and plant) ACC-deaminase, adding another layer of metabolic complexity to the regulation of ACC levels. It is also well established that ACC can be easily transported over short (intracellular and intra-tissue) and long-distances (via the xylem and phloem), providing the plant with an elaborate system to control local and remote ethylene responses. Last but not least, ACC has been identified as a potential signaling molecule, independent of ethylene. This property of ACC is perhaps the most exciting, opening new avenues in ACC research, with potentially profound effects on plant physiology. The molecular mechanism by which ACC is signaling and the identity of other putative signaling components in such an ‘ACC pathway’ remain to be discovered.

## AUTHOR CONTRIBUTIONS

Bram Van de Poel and Dominique Van Der Straeten conceived the topic and wrote the manuscript

## Conflict of Interest Statement

The authors declare that the research was conducted in the absence of any commercial or financial relationships that could be construed as a potential conflict of interest.
